# Emotional Memory in Patients with Alzheimer's Disease: A Report of Two Cases

**DOI:** 10.1155/2012/313906

**Published:** 2012-08-08

**Authors:** Akira Okada, Junko Matsuo

**Affiliations:** Department of Psychiatry, Nara Hospital, Kinki University School of Medicine, 1248-1 Otodacho, Ikoma City 630-0293, Japan

## Abstract

Highly emotional events in daily life can be preserved in memory and such memory is generally referred to as emotional memory. Some reports have demonstrated that emotional memory is also found in patients with Alzheimer's disease (AD). However, to our knowledge, there have been no reports about how long memory retention for emotional events can continue in patients with AD. In this paper, we present two patients with AD who lost an immediate family member during followup and retained the memory over a long period despite progression of the AD.

## 1. Introduction

The study of emotional memory in patients with Alzheimer's disease (AD) takes two general paths. The first involves the use of photographs, words, and stories to investigate delayed recognition memory for emotional stimuli in an experimental setting. Several such studies have found emotional enhancement effects in AD [1–3], but there is some debate over the effects of emotional content on memory [4]. The second involves an investigation of the real-life experiences of patients with AD. One such study looked at experiences of the great Hanshin-Awaji earthquake in Japan in 1995 [5]. The findings revealed that subjects better remembered the earthquake than a magnetic resonance imaging (MRI) examination performed after the earthquake (mean interval of 59.2 days between the earthquake and memory survey versus 17.5 days between the MRI examination and survey). In addition, they recalled personal events during the earthquake well but nonpersonal, factual knowledge poorly. These studies provide findings that are extremely useful to our understanding of AD, but to our knowledge, there have been no reports about how long memory retention for emotional events can continue in patients with AD.

In this paper, we present two patients with AD who lost an immediate family member during followup and retained memory of the event over a long period despite progression of the AD. Both patients' families provided verbal informed consent to publish the cases, with due consideration given to protecting the patients' identities.

## 2. Case Presentation

### 2.1. Case 1

Case 1 was a 75-year-old woman who visited our hospital with her son due to her insistence that her bag had been stolen, but for around two years she had often misplaced her bag or purse. She had no history of psychiatric disorders.

At the initial visit, no abnormal findings were found on routine blood tests, hematochemistry, or electroencephalography. Magnetic resonance imaging (MRI) of the brain showed no cerebrovascular damage, organic change, or striking atrophy. Her mini-mental state examination (MMSE) [6] score was 20 (orientation to time 2/5, orientation to place 2/5, registration 3/3, attention and calculation 5/5, recall 1/3, naming 2/2, repetition 1/1, following commands 3/3, reading 1/1, writing 0/1, and design copy 0/1). We gave a diagnosis of stage 4 AD (mild dementia) using the functional assessment staging (FAST) tool [7]. She was started on donepezil 5 mg/day and returned for follow-up visits with her son about once a month. Just over two years (26 months) after her first visit, she began to use elderly day care services and 17 months later she could not do simple calculations or shop alone. At five and a half years months after the first visit, she travelled abroad with her family, but had no recollection of the trip three weeks after returning to Japan. Then at just over seven years after her first visit, her son died of kidney cancer. Three weeks later, she said, “My son died. I wanted to pass away earlier than him,” but she could not accurately remember factual knowledge about the funeral or Buddhist services she attended (e.g., approximate number of people at the funeral, place of the funeral home, and number of Buddhist services). We rated her at FAST stage 5 (moderate dementia) at this time. Eight and a half years after the first visit, she was assessed to be at FAST stage 6.6 (moderately severe dementia characterized by improperly putting on clothes, difficulty adjusting bath-water temperature, forgetting to flush the toilet, and urinary incontinence) and increased the dose of donepezil to 10 mg/day. Three years later (137 months after first visit), she verbally expressed the same feeling as shortly after her son had died. Two weeks later, she was admitted to our hospital because of loss of consciousness. MRI examination of the brain on admission showed no cerebrovascular damage or organic change but there was enlargement of both lateral ventricles. She died of cardiac failure after two days in hospital.

### 2.2. Case 2

Case 2 was a 65-year-old woman who visited our hospital with her husband because she had developed feelings of anxiety and sleep disturbance after visiting her mother in hospital. She had become unable to cook, make telephone calls, and shop without assistance for two years. She had no history of psychiatric disorders.

At the initial visit, there were no abnormal findings on routine blood tests or hematochemistry. Brain MRI showed no cerebrovascular damage, organic change, or striking atrophy. Her MMSE score was 18 (orientation to time 3/5, orientation to place 4/5, registration 3/3, attention and calculation 1/5, recall 0/3, naming 2/2, repetition 1/1, following commands 3/3, reading 1/1, writing 0/1, and design copy 0/1). We gave a diagnosis of AD, FAST stage 4 (mild dementia). She was given donepezil 5 mg/day and returned for follow-up visits with her husband around once a month. Feelings of anxiety and sleep disturbance showed improvement at the two-month follow-up visit.

Twenty months after the first visit, her mother died. Eighteen days later, she said “I miss my mother. I want someone to be around me.” Like Case 1, she could not accurately remember factual knowledge about the funeral and Buddhist services she attended. Her diagnosis was FAST stage 4 (mild dementia) at this time. Three years after the first visit, she very often forgot to turn off taps and electrical equipment, and medication noncompliance was suspected. Six months later, brain MRI showed no cerebrovascular damage or organic change but did show enlargement of both lateral ventricles. ^123^I-IMP brain single photon emission computed tomography showed symmetrical decreased cerebral blood flow in bilateral temporal and parietal lobes. She was judged to be at FAST stage 6.4 (moderately severe dementia characterized by improperly putting on clothes, difficulty adjusting bath-water temperature, and forgetting to flush the toilet) and donepezil was increased to 10 mg/day. Five years after the first visit, she often experienced nocturnal delirium after nontypical events such as dental treatment. Four months later (64 months after first visit), she was admitted to a special nursing home for the elderly. Ten days before admission, her MMSE score was 5 and she verbally expressed the same feeling as when her mother had died.

## 3. Discussion

In the two cases presented, we noted four interesting findings. First, emotional memory was found to be intact at FAST stages 4 (mild) and 5 (moderate) in our two patients with AD. They expressed feelings of desolation and loneliness at 20 days and 18 days, respectively, after their immediate family member had passed away even though they often forgot notable life events that had taken place just a few days earlier. In general, AD is a slowly progressive disease caused by atrophy of the medial temporal lobe including the amygdala, which is thought to play a crucial role in emotional memory [8]. Therefore, it is speculated that the progression of AD possibly undermines the effects of emotional arousal on memory. Our cases suggest that such effects may not be undermined until beyond FAST stage 5 in patients with AD.

Second, marked emotional arousal was effective for retaining memory of their immediate family member dying but not for retaining factual knowledge about the funeral or Buddhist services. This is consistent with the finding reported for patients with AD that experienced the great Hanshin-Awaji earthquake mentioned above [5].Emotional memory enhancement might therefore have greater effect on episodic memory than on semantic memory in patients with AD.

Third, emotional arousal was caused by an extreme negative event. In Case 1, before the son's death, it had not been triggered by overseas travel, which can be considered to be positive in terms of emotional valence. This suggests that negative real-life events that cause psychological distress, such as losing a family member, might easily produce greater emotional enhancement effects than positive events in patients with AD.

Lastly, we know from experience that a favorable environment is helpful in managing patients with AD. Both of our patients retained memory of their family member's death even at FAST stage 6 (moderately severe dementia), indicating that patients with AD may retain memory for highly negative events over a long period even with disease progression.

In conclusion, these two cases suggest that patients with AD may preserve the ability to store negative events for long periods, until at least FAST stage 5. These findings indicate that memory of experiences in an unfavorable care environment, such as an abusive one, may be retained in the memory of patients with AD. More reports of cases are needed to clarify the nature of emotional memory in AD.
